# Validation of a Biomechanical Injury and Disease Assessment Platform Applying an Inertial-Based Biosensor and Axis Vector Computation

**DOI:** 10.3390/electronics12173694

**Published:** 2023-08-31

**Authors:** Wangdo Kim, Emir A. Vela, Sean S. Kohles, Victor Huayamave, Oscar Gonzalez

**Affiliations:** 1 Ingeniería Mecánica, Universidad de Ingenieria y Tecnologia—UTEC, Lima 15063, Peru; 2 Research Center in Bioengineering, Ingeniería Mecánica, Universidad de Ingenieria y Tecnologia—UTEC, Lima 15063, Peru; 3 Kohles Bioengineering, Cape Meares, OR 97141, USA; 4 Division of Biomaterials & Biomechanics, School of Dentistry, Oregon Health & Science University, Portland, OR 97239, USA; 5 Department of Emergency Medicine, School of Medicine, Oregon Health & Science University, Portland, OR 97239, USA; 6 Department of Human Physiology and Knight Campus for Accelerating Scientific Impact, University of Oregon, Eugene, OR 97403, USA; 7 Department of Mechanical Engineering, Embry-Riddle Aeronautical University, Daytona Beach, FL 32114, USA

**Keywords:** biosensors, instantaneous axis-angle representations, IMU, inertial measurement units, quaternions, inverse and forward kinematics, instantaneous axis of rotation, motion tracking sensors

## Abstract

Inertial kinetics and kinematics have substantial influences on human biomechanical function. A new algorithm for Inertial Measurement Unit (IMU)-based motion tracking is presented in this work. The primary aims of this paper are to combine recent developments in improved biosensor technology with mainstream motion-tracking hardware to measure the overall performance of human movement based on joint axis-angle representations of limb rotation. This work describes an alternative approach to representing three-dimensional rotations using a normalized vector around which an identified joint angle defines the overall rotation, rather than a traditional Euler angle approach. Furthermore, IMUs allow for the direct measurement of joint angular velocities, offering the opportunity to increase the accuracy of instantaneous axis of rotation estimations. Although the axis-angle representation requires vector quotient algebra (quaternions) to define rotation, this approach may be preferred for many graphics, vision, and virtual reality software applications. The analytical method was validated with laboratory data gathered from an infant dummy leg’s flexion and extension knee movements and applied to a living subject’s upper limb movement. The results showed that the novel approach could reasonably handle a simple case and provide a detailed analysis of axis-angle migration. The described algorithm could play a notable role in the biomechanical analysis of human joints and offers a harbinger of IMU-based biosensors that may detect pathological patterns of joint disease and injury.

## 1. Introduction

Human motion capture systems, constructed from Inertial Measurement Units (IMUs), have been the subject of recent development and validation. Lapresa et al. (2022) presented the validation of inertial systems using an anthropomorphic robot [1]. Recent work by Bialecka et al. (2023) used IMUs to validate a robot arm using shoulder range of motion [2]. Perez-Sanpablo et al. (2023) [3] validated IMUs to assess trunk control in subjects with spinal cord injury (2023). Riek et al. (2023) [4] validated IMUs to evaluate gait stability. An IMU system offers several advantages when compared to other motion capture systems. For instance, marker-based optical systems have been the gold standard because of their accuracy when measuring human motion kinematics. However, this type of system requires complex calibration and large operating spaces. They are also expensive and can be affected by reflective objects and occlusion [5]. IMUs are beneficial compared to other motion capture systems because of their low cost, portability, and wearability.

These approaches rely on measuring the three-dimensional linear and angular positions and accelerations of subject joints and limbs generated by micro-electromechanical systems (MEMS) such as an IMU. Effectively, an IMU is a localized biosensor accelerometer and gyroscope that estimates an object’s biomechanical position and orientation. IMUs can be single-point sensors or more complex single-pack arrays when including an additional magnetometer and sensor fusion algorithm, providing more accurate movement data and reduced sensor drift. A common artifact of accelerometer measurements is manifested in velocity and displacement trajectory drift obtained when integrating the raw acceleration record.

MEMS-based IMU sensors can be used in computer vision techniques that track the location of a person through a combination of their pose and orientation with applications in robotics, personal navigation, and virtual reality. Furthermore, recent studies confirm IMU sensor applications for human motion analysis, enhancing biomechanics, rehabilitation, ergonomics, and sports assessments [6]. This research includes refined quantification of human movements and movement classification. These studies concentrate on obtaining the kinematic identification of a particular activity, which helps identify biomechanical disorders such as disease or injury, as well as longer-term patterns of atypical neuromuscular control. Compact, self-contained systems for the kinematic identification of human motion, such as that offered by IMUs, are independent of the subject’s mobility environment and free of obstructions that may affect optical position sensors [7].

Previous work addressing joint biomechanics identified the geometric change to the knee joint’s dynamic alignment as an influence on the stance phase of foot loading [8]. The results also elucidated the mechanical influence of osteoarthritis within knee function when ligaments were situated such that forces acting along them equilibrated during one degree of freedom knee joint constraint [9]. Further characterization of knee motion has been explored in terms of instantaneous joint axes [10] utilizing benchmark data [11] as recorded through a marker-based optoelectronic system (OS). As an enhancement to these approaches, IMUs will commonly package linear accelerometers with angular gyroscopes to identify multiple axes of rotation, where the accelerometer readings may be employed directly without numerical integration [7]. The option of numerical integration may refine the output while introducing additional sensor noise or bias sensitivities. Researchers have also concurrently recorded a biomechanical hinge’s free swing motion using both OS and IMU, concluding that motions measured by IMUs are more precise while the OS motions were more accurate [12].

The present study describes the characteristics of an IMU wearable sensor platform that provides a critical biomechanical parameter during the assessment of joint disease and injury. Here, the instantaneous axis-angle representation (IAA) of limb function is a vector identified as a metric to assist human movement analysis for rehabilitation and sports. The estimation of the IAA and its variant motion is strongly related to the joint’s functionality and ligament health [10] as well as the overall performance of locomotion perception and motor control [13]. Joint kinematics depend on postural balance or equilibrium, meaning that the components of the resultant moment about the axis of rotation sum to zero. In this study, we confirmed the accuracy of IMU-based inverse and forward kinematics as applied to the lower kinematics of an infant dummy and then applied to the upper limb movement of a living subject.

The specific objective of this study is to present a new algorithm for Inertial measurements unit (IMU)-based motion tracking with quaternions. Axis-angle representation for rotation, instead of representing a 3D rotation using a sequence of rotations around the sensor coordinates system, as Euler angles do, the axis-angle representation uses a normalized vector (S) around which the rotation is defined by some angle (θ) and can track a sequence of events in terms of a one-one correspondence of IAA. Although the IAA is not fixed, it is indeed moving about in such an intricate way that has unity relative to the posture and behaviors of the subject being considered.

There are two main advantages to using the axis angle representation for describing limb kinematics. The first is that they allow a global description of rigid body motion that does not suffer from singularities due to local coordinates. Such singularities are inevitable when one represents rotation via Euler angles. The second advantage is that the axis-angle provides a very geometric description of rigid motion, which significantly simplifies biomechanism analysis and is handy for describing the kinesthesis, “feeling of movement,” in all skeletal and muscle structures. The axis vector is not moving instantaneously, occupying a stationary axis in the global frames.

## 2. Materials and Methods

### 2.1. Inertial Measurement Unit Device and Model Foundation

The newest generation of cost-efficient inertial motion trackers features a lightweight design, wireless connectivity (Bluetooth Low Energy, BLE (Nokia, Karaportti, Finland)), and robust sensor fusion algorithms to provide accurate data for human movement applications (DOT Wearable Sensor, Xsens Technologies B.V., Enschede, The Netherlands). Software manipulation tools are provided (Software Development Kit, SDK Movella DOT 2023.6) to facilitate the customization of mobile applications based on the available output data, thereby allowing developers to integrate the sensor into a wide range of solutions. Robust algorithms (Strap Down Integration, SDI) and a sensor fusion framework (Xsens Kalman Filter Core, XKF) run onboard the sensor to provide accurate physical orientation estimates and minimize the effects of magnetic distortion [6].

**Figure 1.**
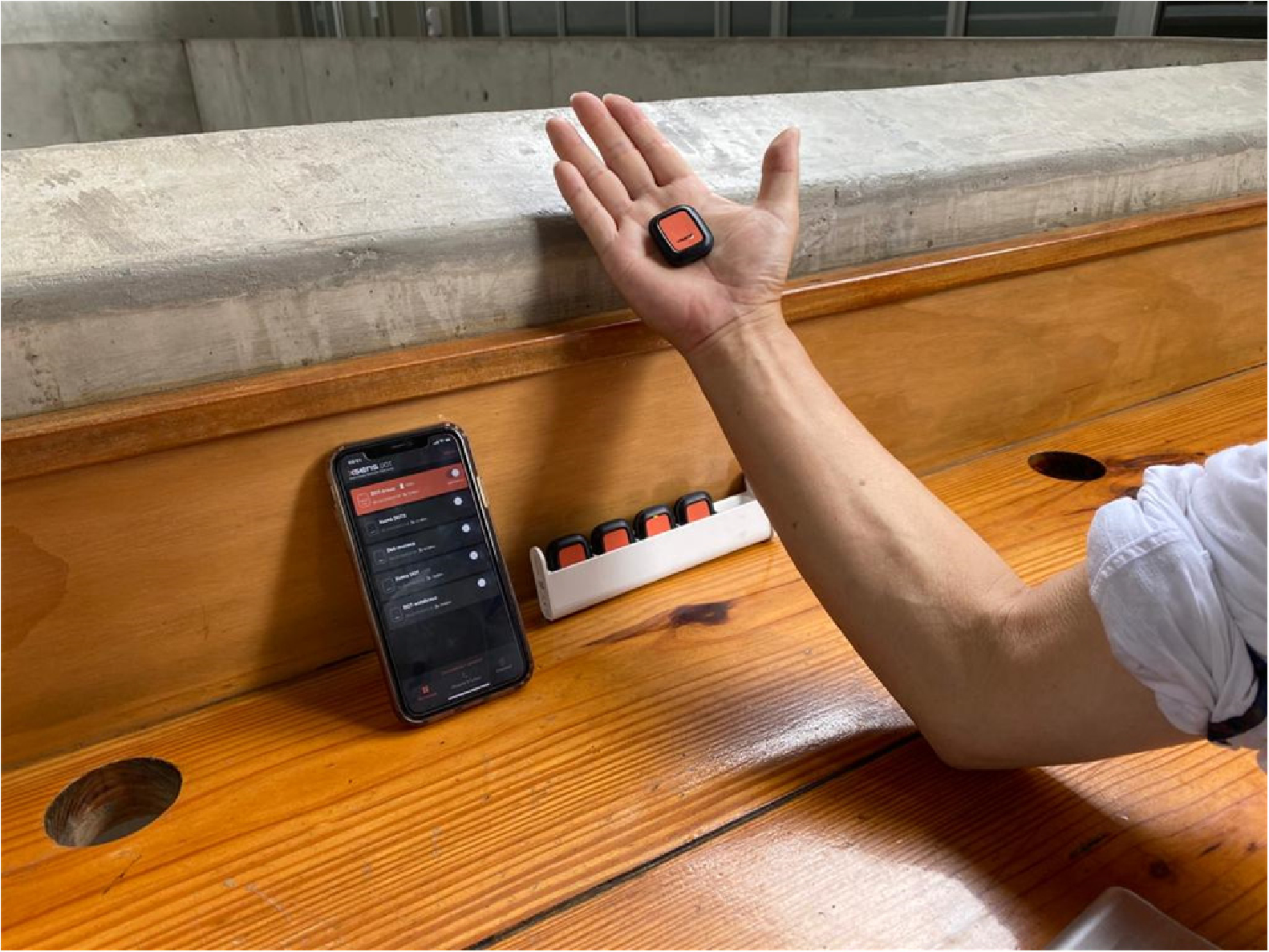
Wireless communication between the sensors and a mobile device was used in this study.

The IMUs applied in this work contain MEMS-type gyroscopes, accelerometers, and magnetometers. These individual sensor signals are fused through a statistical estimation framework to obtain three-dimensional (3D) limb and joint orientation. The output provided by the three main device components is then fed into the signal processing pipeline. The two main algorithms noted above are run onboard the motion tracking sensor [14]. The sensors are primarily designed to connect to mobile devices such as smartphones that must be BLE-capable (Figure 1). The wearable device proposed for use in experiments integrates smart sensors to track a user’s physical behavior, specifically the gyration and orientation of their hand, in high spatial and temporal resolution. This enables real-time multi-parameter tracking as a significant wearable sensor system [15].

**Figure 2.**
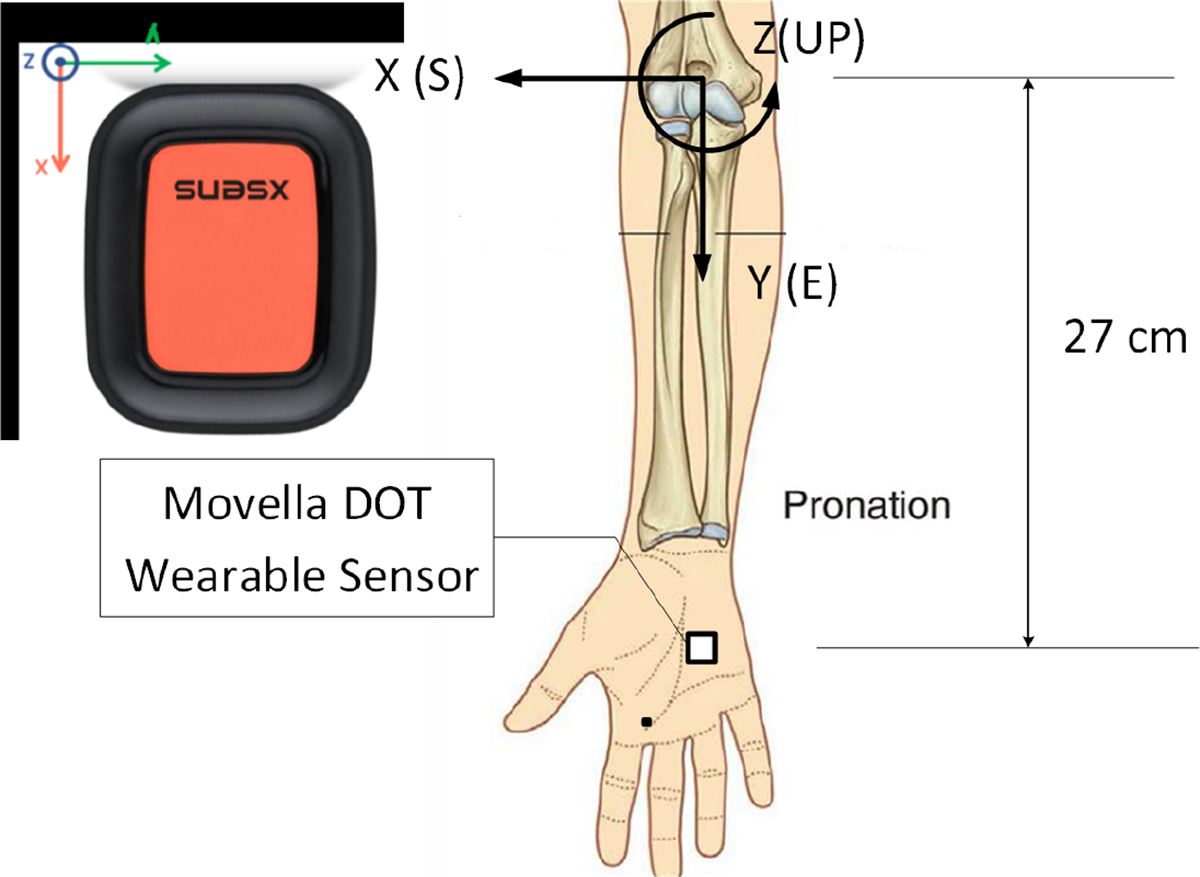
The local sensor coordinate system (SCS) is associated with the sensor as indicated by the (x,y,z) cartesian coordinate system, while the global reference coordinate system (GRCS) is matched to the elbow joint and anatomic orientation as indicated by the (X,Y,Z) cartesian coordinate system. Since the SCS is not aligned with the GRCS in this anatomic configuration, the data measured by the SCS is transformed through vector algebra by applying the unit quaternion $\left[cos\frac{\pi }{4},\;0,\;0,sin\frac{\pi }{4}\right]$, which rotates data in SCS by 90 degrees about the Z axis.

Before describing the output data, the different types of reference systems used in the study are presented. Data shall be expressed in terms of local (Sensor Coordinate System, SCS) and global, earth-fixed (Global Reference Coordinate System, GRCS) coordinate systems. The SCS is a right-handed, cartesian coordinate system that is body-fixed within each sensor identified with lowercase x, y, and z axes (Figure 2). The local earth-fixed GRCS is also defined as a right-handed, cartesian coordinate system identified with uppercase X,Y, and Z axes with the following global orientations:

- X positive to the East (E).
- Y positive to the North (N).
- Z positive when pointing Up (U)

This coordinate system is known as East-North-Up (ENU) and is the standard framework in inertial navigation for aviation and geodetic applications. Note that positive global orientations can be established for any application while maintaining the right-hand configuration, i.e., X positive to the South (S).

The wearable sensors produce instantaneous 3D coordinate axis orientation and acceleration data. The data available for the experiment can be classified into two categories: inertial data and sensor fusion data. The inertial data is comprised of linear acceleration (units of m/s^2^) and angular velocity (units of ∘/s) as provided in the SCS. These IMU-based sensors output angular velocities as a direct measurement from the internal gyroscopes. The 3D orientation output takes the quotient of the axis vectors as unit quaternions. The orientation can be represented by a normalized quaternion, q=[WXYZ], with W being the real component and X, Y, Z as the imaginary global coordinate components. This sensor output is within the ENU localized global reference coordinate system. The output IMU measurement vector $S_{raw}$ contains the individual measurements stacked together as ten state variables:

(1) $$S_{raw}=\left[a_{x},a_{y},a_{z},\omega_{x},\omega_{y},\omega_{z},q_{0},q_{1},q_{2},q_{3}\right]$$

where a represents the linear acceleration, w represents the angular velocity in the sensor’s local coordinate system, and q represents the quaternion. The optimal filtering problem is then to determine the angular acceleration state variables, $\left[\alpha_{x},\alpha_{y},\alpha_{z}\right]$ as well as their numerical derivatives as the angular velocity vector components $\left[\omega_{x},\omega_{y},\omega_{z}\right]$. Further, the problem is then constructing the new state variables, which provide the best match with the data within $S_{raw}$ but also have a degree of numerical smoothness. The regularization method is then applied to solve this numerical challenge [16].

In this study, we estimated the identified state variables by applying L-curve Tikhonov regularization filtering (TRF). The TRF algorithm was previously applied in the optimization of smoothing parameters [17] during multiscale cell-tissue level [18] and joint level [19] biomechanical analyses.

As a result of the TRF, thirteen numerically smoothed state variables are then present in the filtered vector $S_{smooth}$:

(2) $$S_{smooth}=\left[a_{x},a_{y},a_{z},\omega_{x},\omega_{y},\omega_{z},q_{0},q_{1},q_{2},q_{3},\alpha_{x},\alpha_{y},\alpha_{z}\right]$$

The data in this application were then recorded through local resources (VR Motion Laboratory, Department of Mechanical Engineering, UTEC, Lima, Peru). One healthy male, well-trained subject provided his written informed consent to participate in this study.

It has frequently been assumed in previous methods that the point of observation for motion is unoccupied because it is measured in a SCS, while the point of observation in this work is occupied in GRCS. When a point of observation is occupied, there is also information to specify the motion of the subject, and the limb of the person in action instantaneously occupies some portion of the space in a way that is unique to the person as presented as the instantaneous axis-angle representation (IAA.) This information is unique to that person. The IAA is not moving but stationary in the GRCS, occupying the specific axis in the freedom space. Therefore, the innovation brought by this research is to propose the measure of the feeling of the self-movement, i.e., proprioception, in terms of the IAA meaning that it specifies the self-movement as distinguished from an object moving in the environment.

### 2.2. Inverse Kinematic Solutions Using Quaternions

Our solution method is based on an axis-angle representation by applying vector algebra quaternions as a motion operator. All rotating screw motions are represented as a rotation about an axis with respect to the global GRCS. Two quaternions describe general movement positioning: one for orientation and the second for translation.

All the data processing was implemented in a commercial programming and computing platform (MATLAB, The MathWorks, Natick, MA, USA). Here, the module “Quaternion.m” was applied [20]. Quaternion.m implements quaternion mathematical operations, including three-dimensional rotations, transformations, and numerical propagation of the governing equations of rotational motion, most of which are fully vectorized.

Quaternions represent complex numbers within a four-dimensional vector space (rank 4) over a real number field [21]. A quaternion is generalized as

(3) $$q=w+xi+yi+zk=\left(q_{0},q_{1},q_{2},q_{3}\right)$$

or

(4) $$q=\left(q_{0},q_{v}\right)$$

where $q_{0}$ represents a scalar and $q_{v}=\left(q_{1},q_{2},q_{3}\right)$ represents a vector. A quaternion of $q_{v}=0$ is called a real quaternion, and a quaternion of $q_{0}=0$ is identified as a pure quaternion. Multiplication of two quaternion vectors can be expressed as

(5) $$q_{a}⊗q_{b}=q_{a0}q_{b0}-q_{av}\cdot q_{bv},q_{a0}q_{bv}+q_{b0}q_{a0}+q_{av}\times q_{bv}$$

where the symbols “⊗”,“⋅”,“×” denote the quaternion product, dot product, and cross product actions, respectively. Quaternion multiplication is not considered commutative.

The conjugate of the quaternion can be expressed as:

(6) $$q^{*}=\left(q_{0},-q_{v}\right)=\left(q_{0},-q_{1},-q_{2},-q_{3}\right)$$

and thus, defining the quaternion norm ∥q∥ as:

(7) $$|q{|}^{2}=q⊗q^{*}=q_{0}^{2}+q_{1}^{2}+q_{2}^{2}+q_{3}^{2}$$

with the relationship ${\left|q\right|}^{2}=1$, a unit quaternion is present whereby any quaternion (q) can be normalized by dividing by its norm. The inverse of a quaternion is then expressed as:

(8) $$q^{-1}=\frac{1}{{∥q∥}^{2}}q^{*}\;\text{and}\;∥q∥\neq 0$$

and thereby for a unit-quaternion, the relationship is reduced to:

(9) $$q^{-1}=q^{*}$$

**Figure 3.**
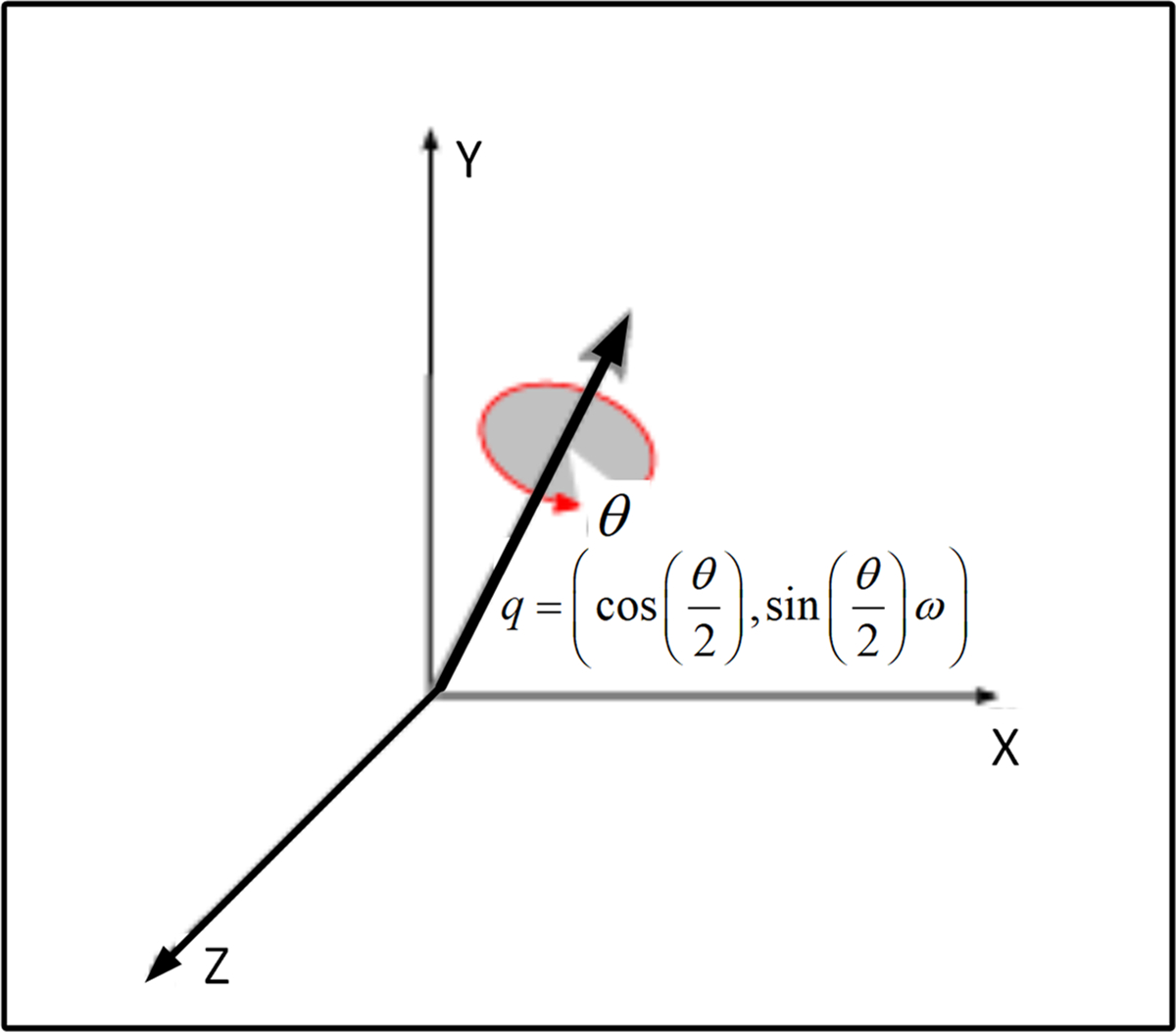
Graphical axis-angle representation for vector rotations. The approach described here uses a normalized quaternion q around which the rotation is defined by four kinematic variables instead of three. Applications include computer-aided graphics, vision, and virtual reality computation.

A unit quaternion can be further defined as a vector rotation operator. Rotation about a unit axis ω with an angle θ is then defined by the axis-angle representation (Figure 3)

(10) $$q=\left(cos\left(\frac{\theta }{2}\right),sin\left(\frac{\theta }{2}\right)\omega \right)$$

### 2.3. Biomechanical Orientation Tracking with Quaternions

Earlier work has demonstrated how human perception and motor control interact continuously with external physical systems [22]. The axis-angle representation effectively establishes a global description of the individual as a rigid body during environmental interactions and avoids mathematical singularities due to the use of the local coordinates. The benefits of using quaternions during axis-angle representation, as described in the presented approach, are the well-defined sets of operations for vector addition, multiplication, and interpolation while converting the representations directly to rotational matrices. Such singularities are inevitable when representing rotations traditionally via Euler angles.

A general rigid-body transformation has 6 degrees of freedom (DOF) accounting for linear and angular translations or as defined here: 3 DOF for orientation and 3 DOF for translation. A unit-quaternion can be used as a rotation operator as shown in Equation (10) and Figure 3.

A vector v can be transformed into a vector w such that:

(11) $$w=q⊗v⊗q^{*}$$

where q is a unit quaternion and v is a pure quaternion. The unit quaternion can be used to transform a vector, but not through rigid transformation. Therefore, an alternative quaternion will implement translation:

(12) $$t=p-q⊗p⊗q^{*}$$

where p is the position vector of an arbitrary point on the axis within a pure quaternion.

In this application, we use the axis-angle representation to obtain the inverse kinematics solution of the kinematics of the dummy and then apply it to the elbow during simple flexion and extension within the healthy range of motion of upper limb movement. The immediate objective is to identify the forward kinematics of the hand. For this purpose, it suffices to identify the values of the axis-angle of the elbow joint and its location with respect to the GRCS system. The estimation of the IAA representation of limb movement, also known as a biomechanical screw axis [23], can play a notable role in the biomechanical analysis of biological joints (healthy, diseased, and injured). We assume that the amplitude of the angle of the instantaneous axis is minute, in conformity with a small angle assumption when combined in the same manner as force values.

### 2.4. Instantaneous Axis Angle Origin Location Algorithm

Poor accuracy and precision when determining the IAA origin obtained from the IMU data are typically due to the lack of a consistent ground reference system within the sensor [12]. Therefore, it is necessary to align the SCS to the GRCS to allow the IAA to be operated on by the quaternions.

In the proposed algorithm, when estimating the IAA origin, the IAA direction s may be identified within the GRCS frame as the positive direction of the resulting angular velocity vector. The screw axis direction can then be readily identified from a gyroscopic rate reading:

(13) $$s=\frac{\omega }{∥\omega ∥}$$

Identifying the screw axis location vector $s_{0}$ then follows from the different sensor measurements. Previously, the origin of the IAA of the knee joint was determined based on the relationship with ground reaction forces during contact while physical posture was treated as a covariant [24]. Alternatively, an average linear distance between geometric points or the midpoint between two anatomic landmarks and the s in Equation (13) can be used as an origin of the IAA. Here, the midpoint of the projection of the center of the medial and lateral aspects of the forearm and humerus was used (Figure 2)

**Figure 4.**
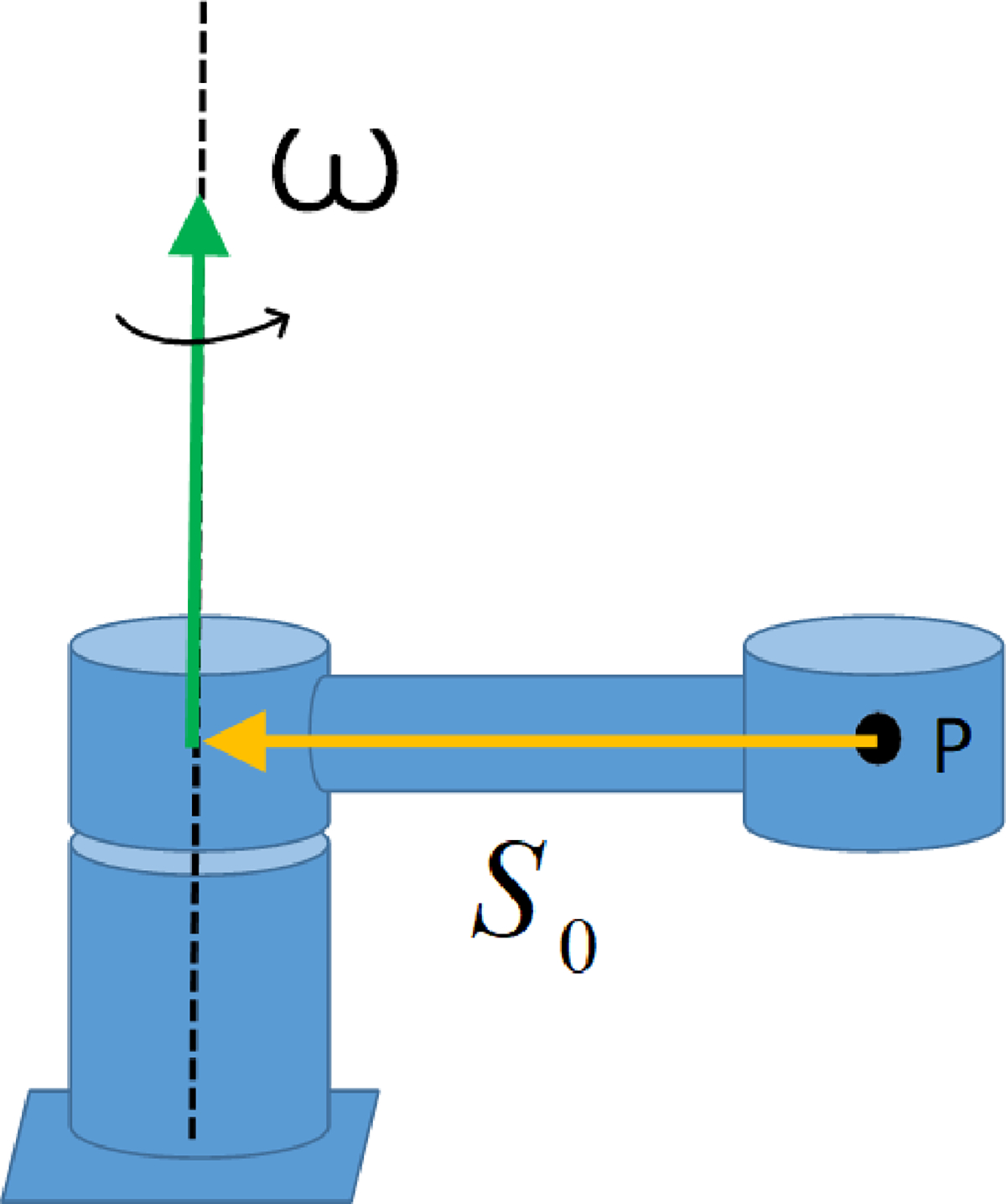
A simplified upper limb with point P is identified at the palmar surface of the hand with rotation about the shoulder joint and free accelerations, referred to as the RC frame [25]. Depending upon the limb’s orientation, the local acceleration due to gravity is subtracted directly from the IMU reading measured in the SC frame.

More systematically, an IMU-based kinematic manipulator identification algorithm can be used [7], where an identified point’s rotation about a fixed axis is considered. Here, a simplified limb experiences joint rotation with angular velocity ω and angular acceleration α (Figure 4). Identification of the IAA location vector $s_{0}$ follows from linear acceleration measurements. By substituting $r=s_{0}$ into the expression:

(14) a=α×r+ω×ω×r

which was derived in the rotation about a fixed axis for linear acceleration a, an IMU located at point P will experience the translational acceleration defined as:

(15) $$a=\alpha \times s_{0}+\omega \times \omega \times s_{0}$$

Here, linear acceleration a is known with the desire to solve for $s_{0}$. Conveniently, this is a linear system of equations and may be written as follows:

(16) $$a=\alpha \times s_{0}+\omega \times \omega \times s_{0}\;\;\to a=[\alpha \times +\omega \times \omega \times ]s_{0}=Ms_{0}$$

The mathematical arrangement is a skew-symmetric matrix representation of the vector cross product where M is a 3 × 3 time-varying matrix. This formulation may be constructed at any given time from the angular velocity vector ω (IMU output) and the angular acceleration vector α (approximated from the finite differential of ω).

Using Equations (11) and (12), a transformation of the end effector (palmar surface IMU) can be given by quaternion $q^{\left(t\right)}$ as the set of quaternion values from the current time step (t), where $q^{\left(t-1\right)}$ indicates the values at the previous time step such that:

(17) $$q_{0}^{\left(t\right)}=q^{\left(t-1\right)}⊗s_{0}^{\left(t\right)}⊗q^{(t-1{)}^{*}}-q^{\left(t\right)}⊗s_{0}^{\left(t\right)}⊗q^{(t{)}^{*}}+q_{0}^{\left(t-1\right)}$$

where $q^{\left(t\right)}$ and $q_{0}^{\left(t\right)}$ indicate rotation and translation quaternions, respectively. The position of the end effector (*ef* subscript) can be given by:

(18) $$q_{ef}^{\left(t\right)}=q^{\left(t\right)}⊗p_{ef}⊗q^{(t{)}^{*}}+s_{0}^{\left(t\right)}$$

As described above, the IMU consists of three linear acceleration sensors and three rotational rate gyroscopes with the transformation from the SCS frame to the GRCS frames.

A mathematical-specific aim satisfied in this line of research is to geometrically view the quaternion operator’s vector-frame action characterized by Equation (11). Viewing the resultant final relationship between the input vector v, the output vector w, and the coordinate frame with a standard orthogonal basis (i,j,k) is supported by adopting either of the following two distinctively different perspectives.

The first perspective is through observations fixed with respect to the coordinate frame (i,j,k). Here, the quaternion operator rotates the vector v about the IAA and through an angle θ. From this perspective, it is convenient to think of the coordinate frames as being fixed while the vector is rotated, often called a *point* rotation.

The second perspective is that observations are made with respect to the fixed vector v. Here, the quaternion operator (Equation (11), $w=q⊗v⊗q^{*}$) rotates the coordinate frame (i,j,k) about the IAA through an angle -θ. From this second perspective, the vector v is fixed while the coordinate frame is rotated, often called a frame rotation. We will apply the Equation (11) operator and interpret this approach geometrically.

## 3. Results

**Figure 5.**
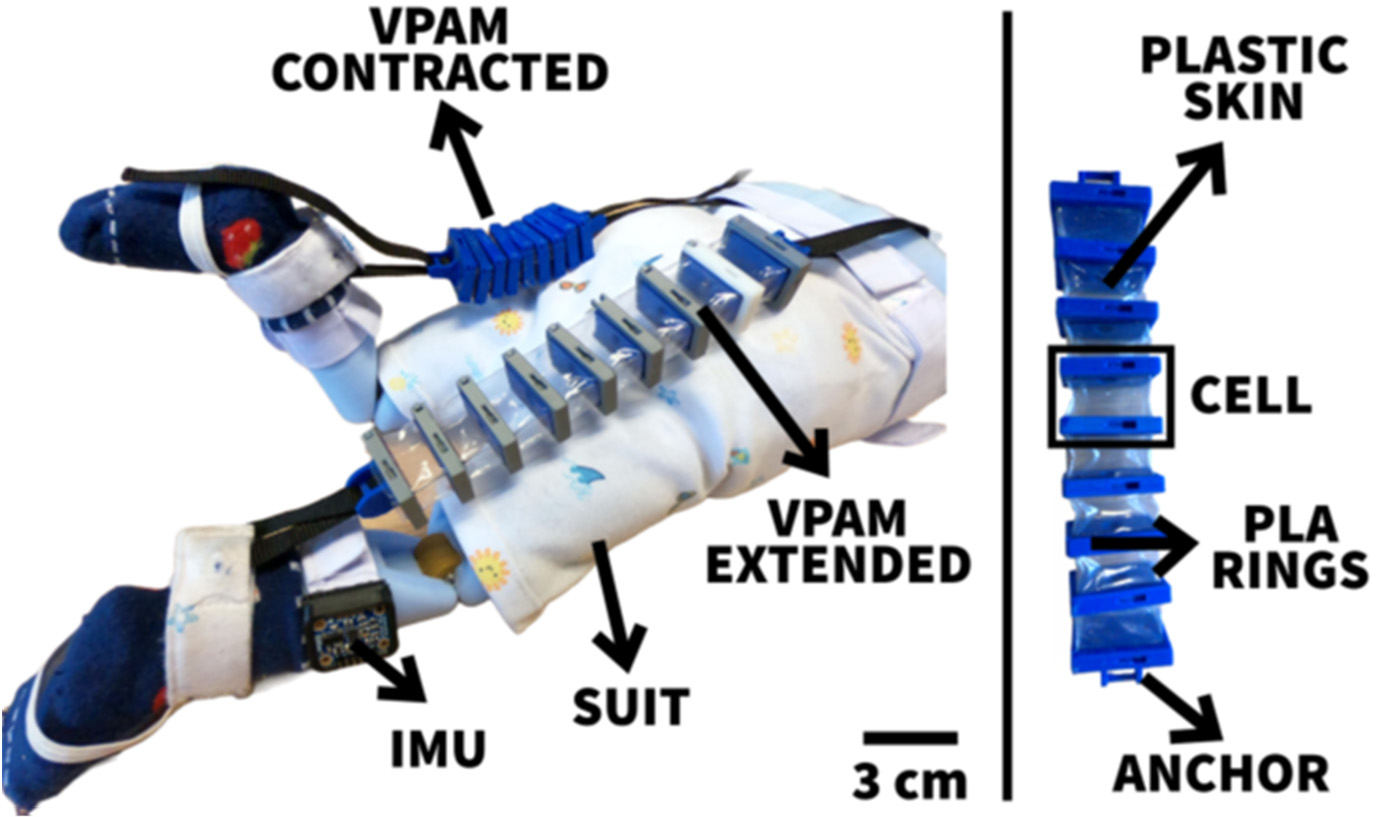
An infant dummy was used for the experimental validation test with vacuum-powered artificial muscles.

**Figure 6.**
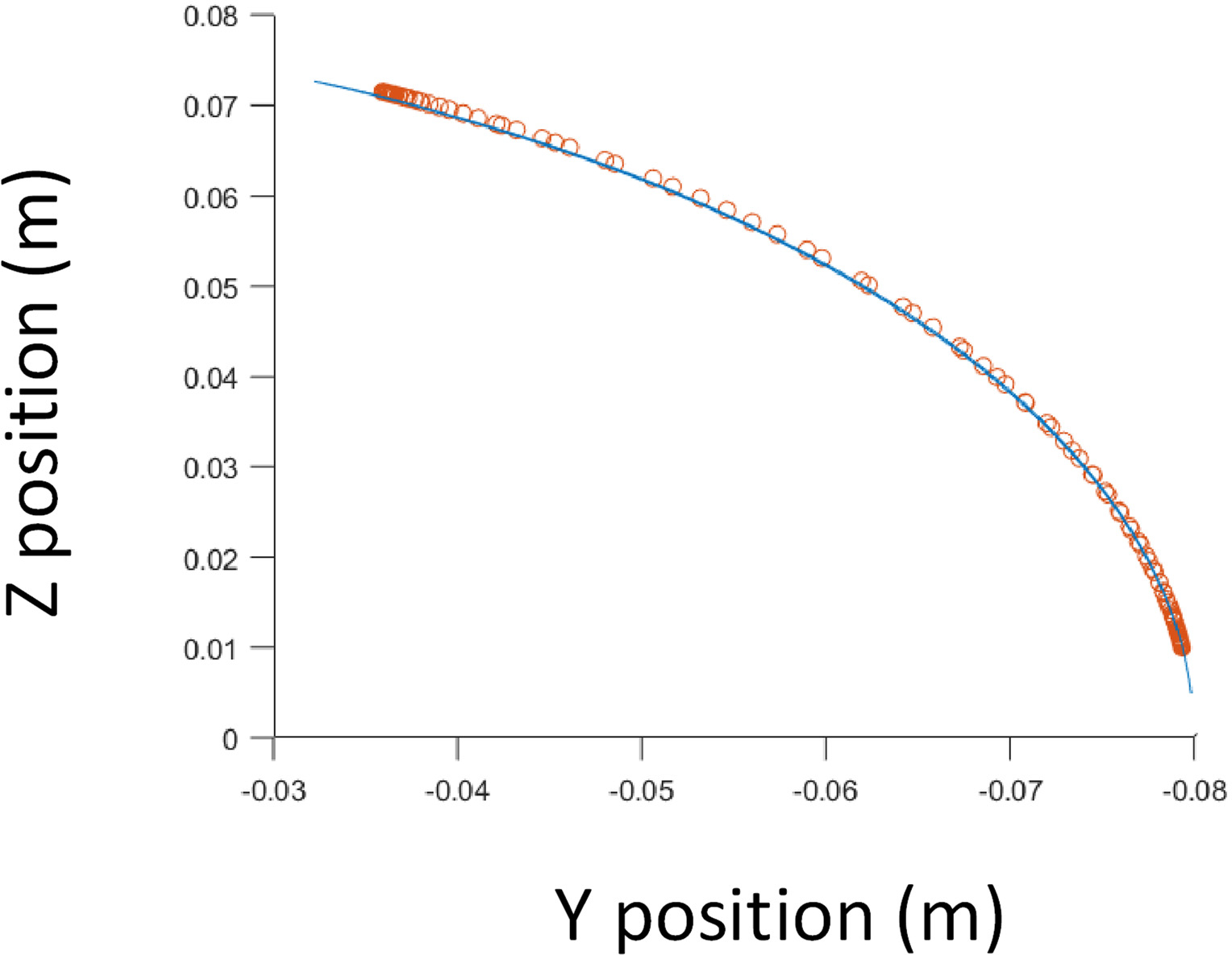
Comparison of trajectories between the quaternion model (blue, sampling rate 60 Hz) and determined data (red, sampling rate 10 Hz) for the dummy model.

Experimental validation of the proposed method is carried out through soft exoskeletons on a dummy model. To validate our approach, the authors utilized lower limbs while modeling was conducted using upper limbs due to data availability. It is assumed that the motion tracking methodology can be applied to any joint [26,27]. These soft exoskeletons consist of wearable garments with active mechanisms to support user motions on different body parts [28]. The objective was to generate controlled and repeatable angular trajectories, thus providing the flexion-extension angles of an infant dummy leg produced by a vacuum-powered artificial muscle simulating a knee flexion-extension-controlled motion (Figure 5). To characterize the various joint behaviors covering different applications, the end effector location predicted by the quaternion model was compared against the determined angle of inclination of the leg. It is clear from the inspection of Figure 6 that the kinematic prediction of this algorithm correlates strongly with the actual end effector location.

Next, we demonstrate the described IMU-based approach by applying the axis-angle representation of healthy upper limb movements. The authors used lower limbs to validate our approach, while modeling was conducted using upper limbs due to the availability of data. It is assumed that the motion tracking methodology can be applied to any joint based on previous studies [26,27]. These experiments: (i) demonstrate the calculation of the IAA and the analysis of IAA migration using quaternion operators; (ii) check the accuracy of both the IMU-based inverse kinematics and forward kinematics. A single male adult subject was used to validate the mathematical approach with functional anatomic data produced during elbow flexion-extension postures of the upper limb within the sagittal plane of motion. The data collection for this study was approved by the UTEC’s human-subjects ethics committee.

The forward kinematics was performed in that sensor trajectories were reconstructed as x, y, and z cartesian coordinates with the help of the IAA. The GRCS frame was defined for the elbow joint as follows: The origin location is the midpoint of the projection between the medial and lateral bony aspects of the distal humerus (Figure 2). The X-axis defines the lateral aspects of the elbow joint. The X-axis is also coincident with the South (S) orientation according to the ENU global reference coordinate system, the Y-axis is positive to the East (E), and the Z-axis is positive when pointing up (U).

Data were generated by the IMU accelerometer and gyroscope, as provided in the SCS, and combined through a sensor fusion algorithm measuring the orientation with respect to the GRCS. Therefore, it was necessary to align the SCS frame in which three linear accelerations and three rotational rate gyroscopes were measured to the global coordinates as described above, allowing the IAA to be computed by the global system.

To visualize the inverse kinematics as a line representation of the IAA in space, we identified the line geometry as determined by its direction and a point on the line itself. We can write the vector equation of the line as:

(19) $$S_{m}=S_{0}\times S$$

which is the moment of the line about the origin 0. Expanding this equation leads to:

(20) $$S_{m}=\left|\begin{array}{ccc} i & j & k\\ x & y & z\\ L & M & N \end{array}\right|=Pi+Qj+Rk$$

**Figure 7.**
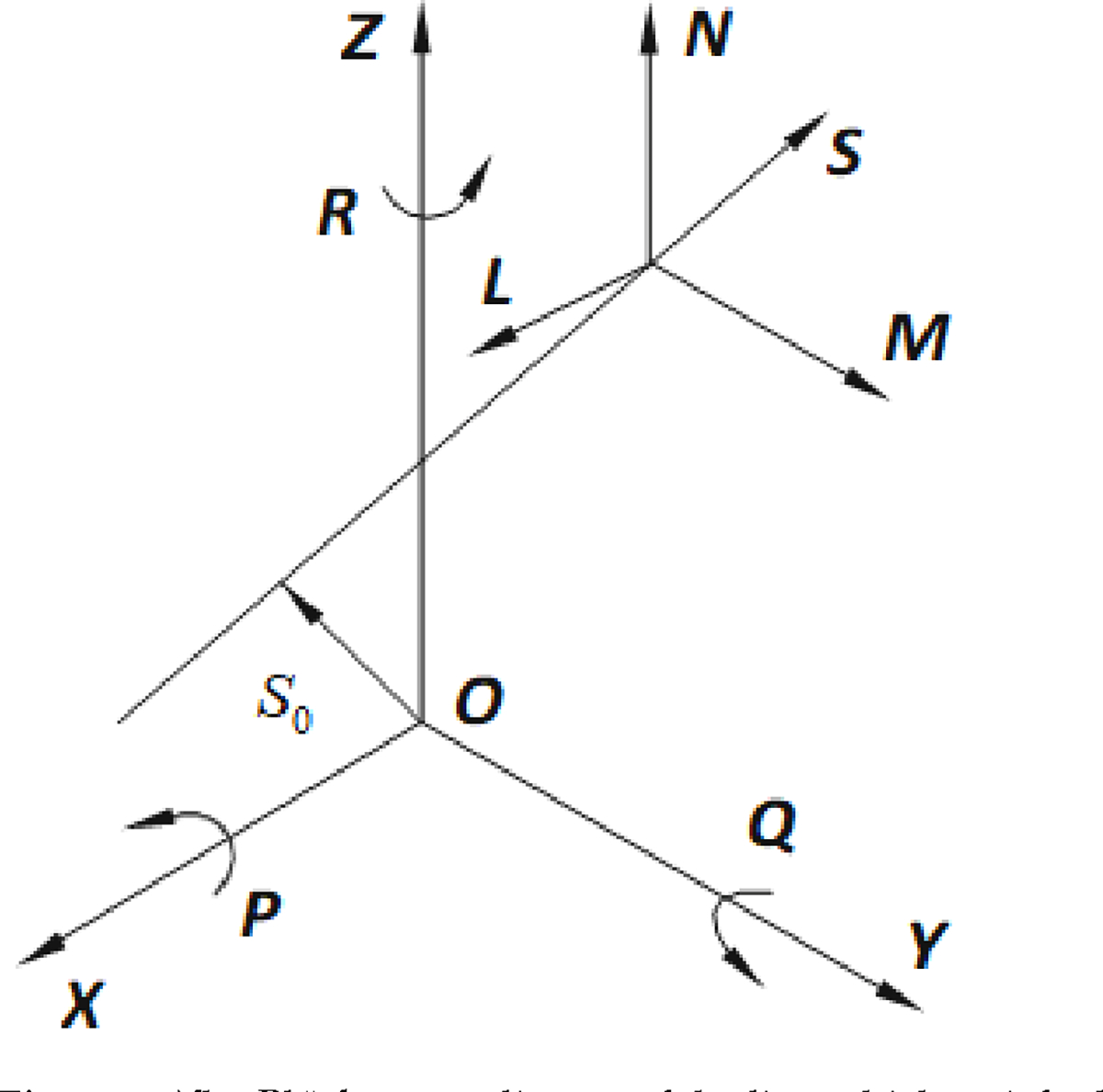
The Plűcker coordinates of the line which satisfy the coordinates $\left(S,S_{0}\right)$.

The orthogonality condition can then be written as:

(21) LP+MQ+NR=0

with the six Plűcker coordinates of the line (L,M,N;P,Q,R) as illustrated (Figure 7).

The (L,M,N) consists of the direction of the line, and (P,Q,R) are the x, y, and z cartesian components of the moment of the line about the origin O.

Further, a given line (L,M,N;P,Q,R) passes through a given plane (t,u,v,s) in the point whose coordinates are defined as:

(22) $$\left[\begin{array}{cccc} t & u & v & s\\ 0 & -N & M & P\\ N & 0 & -L & Q \end{array}\right]\left[\begin{array}{c} x\\ y\\ z\\ w \end{array}\right]=\left[\begin{array}{l} 0\\ 0\\ 0 \end{array}\right]$$

We used a commercial programming platform as previously noted (MATLAB NULL Operation) to solve for the coordinates [x,y,z,w]. Visualization of the results was based on several models, including intersecting pathways of the IAAs within a virtual sagittal plane [29]. This approach extended the previous work describing the embedded kinematics of human joint motion during locomotion [30] including the control of skilled manipulation [31].

**Figure 8.**
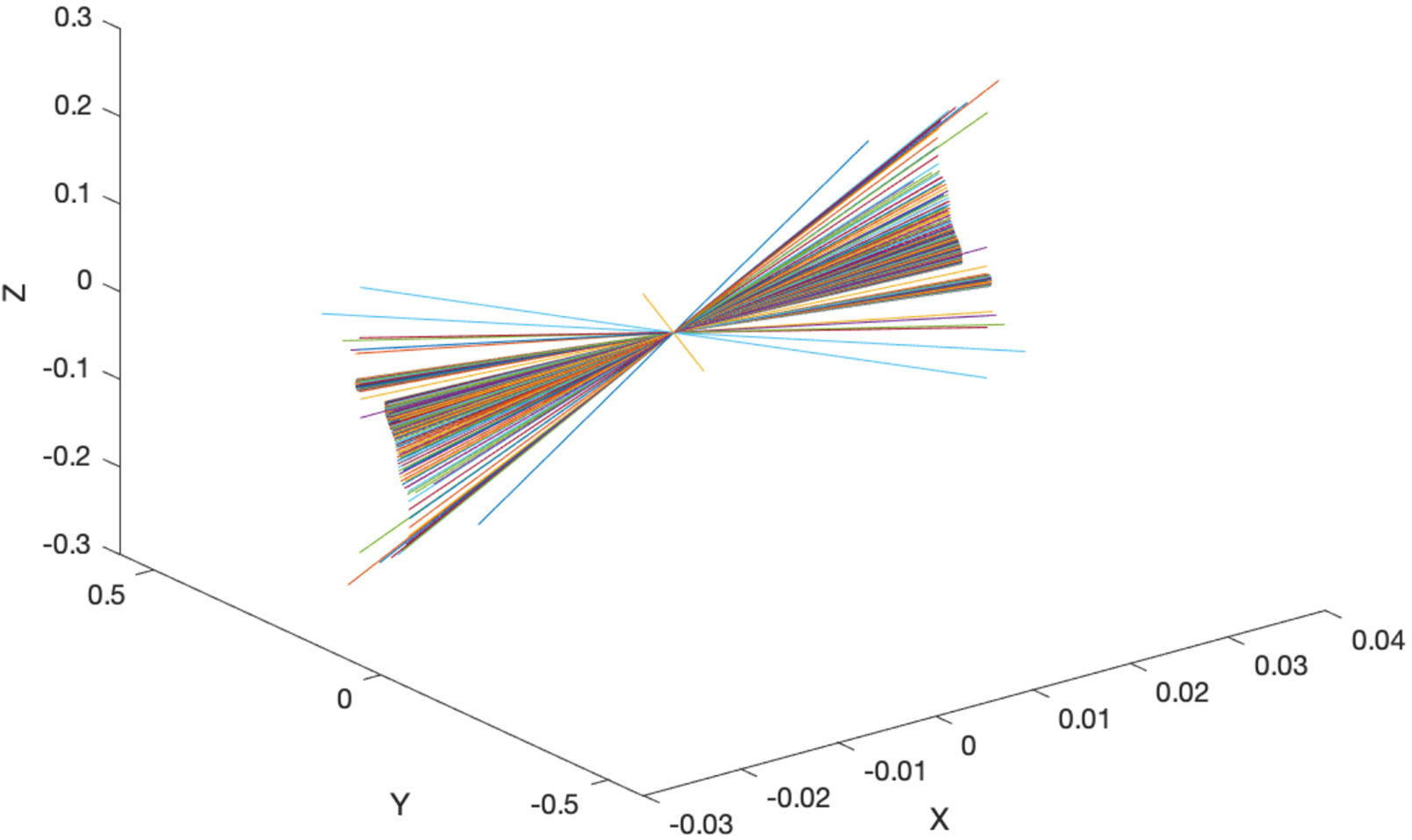
A sequence of IAAs from the single human subject elbow is represented relative to the origin of the global frame to show that IAAs occupy different colors of lines (units in meters, m).

The virtual sagittal plane was defined relative to the geometric representation of the IAA from the geometric center (Figure 8). This allowed the variability assessment in the direction of the functional IAA during the flexion-extension movement of the forearm. In addition, the intersection of the functional IAA with this plane was analyzed, while the migration of IAA was observed for small motion steps (acquired at 60 Hz).

When comparing the Euler angle procedure with the Axis angle procedure, Euler angles tried to force the body to move along the certain route that it had arbitrarily chosen but which the body had not chosen. In fact, the body would not take any one of its routes separately, though it would take all of them together in the most embarrassing manner-goal-directed behavior. The axis angle procedure had no preconceived scheme as to the nature of the movements to be expressed. A subject simply found the body in a certain position, A, and then he coaxed the body to move, not in this way or in that particular way, but any way the body liked to any new position B.

**Figure 9.**
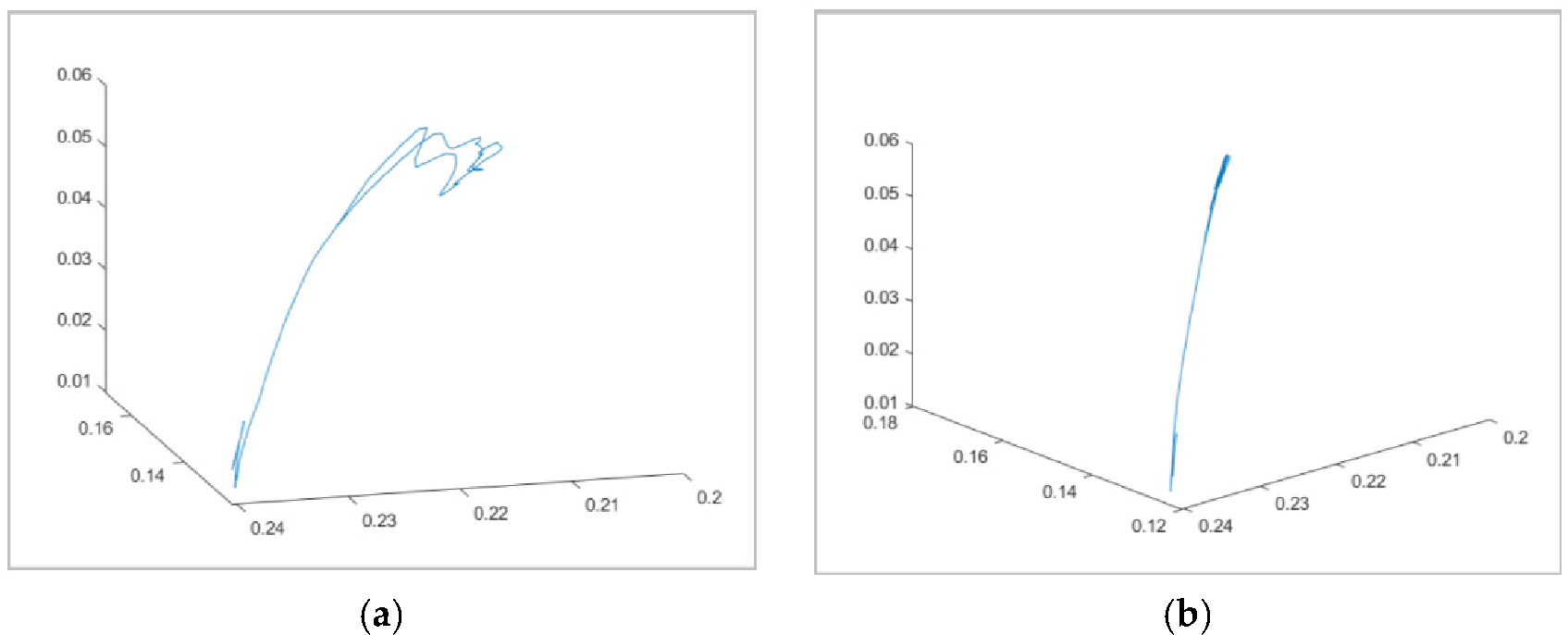
The elbow IAA trajectories as viewed at the oblique angle (**a**) and the sagittal plane containing the motion of flexion and extension (**b**). (units in meters, m).

Once the IAA was defined, forward kinematics obtained the vector trajectories (Figure 9). Finally, the position of the end effector, the IMU sensor located in the palm of the subject’s hand, is given by trajectories as viewed in the oblique and on the sagittal plane.

**Figure 10.**
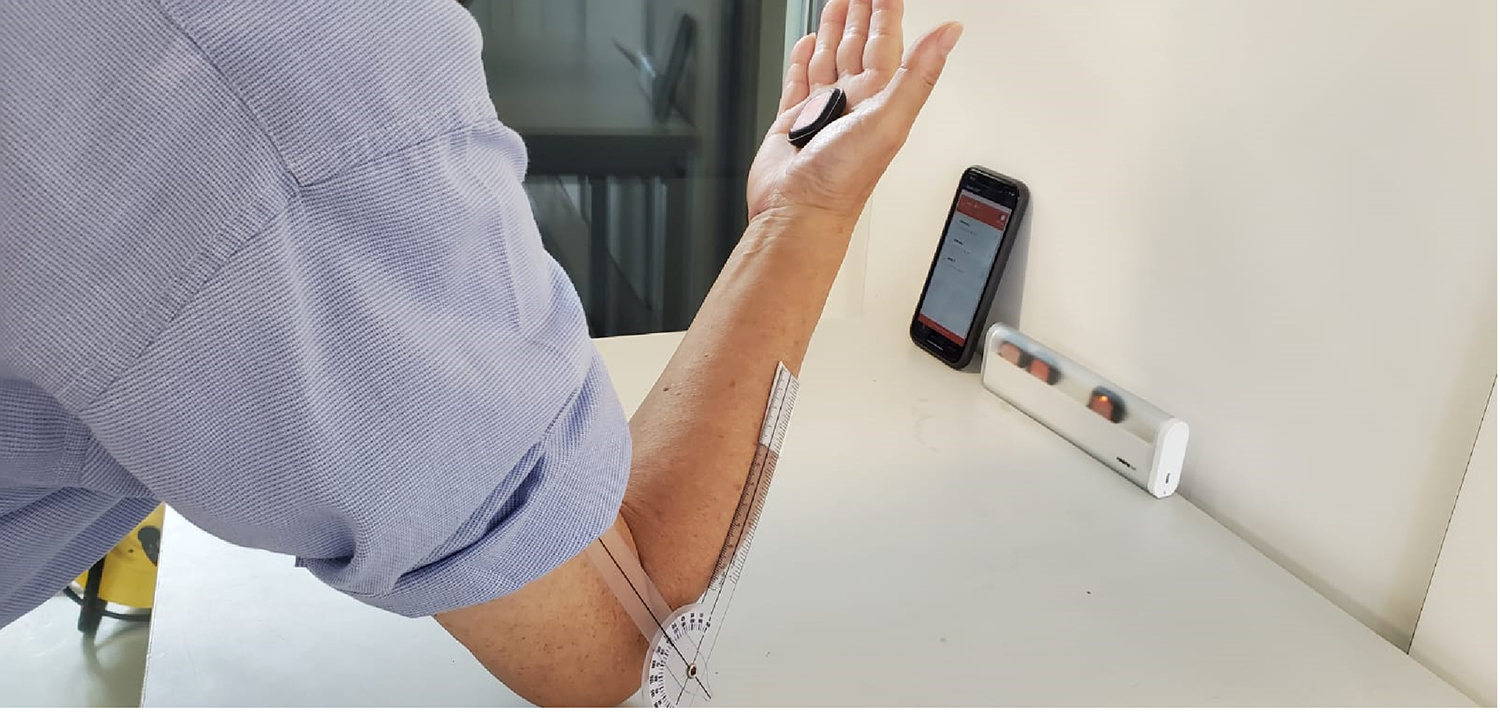
Experimental procedure for angle measurements in two dimensions compared with the angles obtained from the IMU system. The vertical arm of the compass (in white) was fixed onto the lateral side of the upper arm. In contrast, the horizontal arm was mobile to perform accurate 180° amplitude rotations between two segment movements. IMUs and goniometer system coordinates are presented in red and dark grey, respectively. The errors between the two systems are a range of 5% errors.

**Figure 11.**
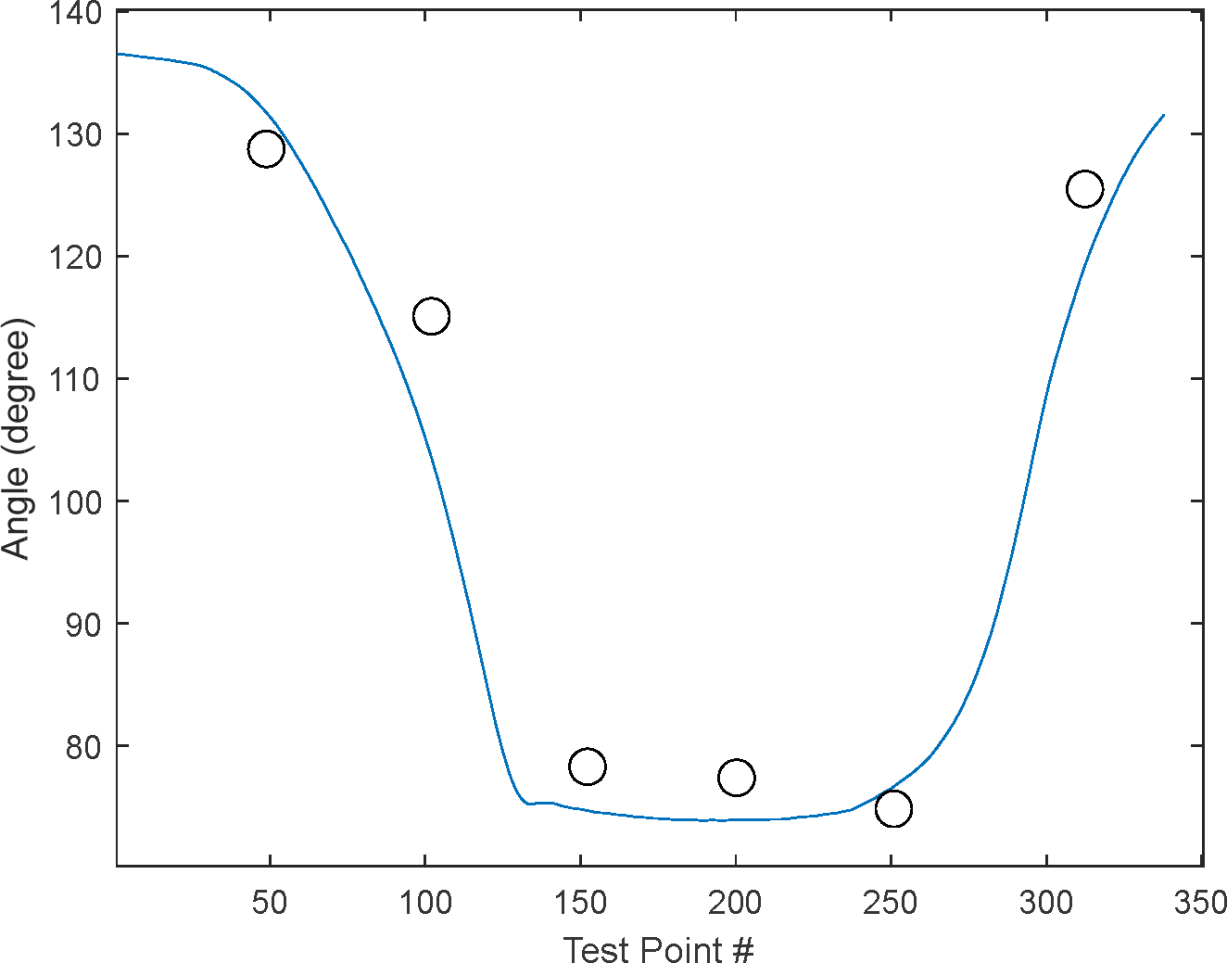
The resulting angle differed by approximately 11% on average versus measurements taken by compass (circle).

In the present study, we computed 2D angular measurements obtained from IMUs relative to those obtained from compass systems; compared to IMU angular measurements (Figure 10), the readings from the goniometer have an error rate of approximately 11% (Figure 11).

## 4. Discussion

Theoretically, for our study, the inverse kinematics, which is ruled by an IAA, is used to visualize the migration of biomechanical action Another piece of information provided by forward kinematics as ruled by the end effector is formed by visualizing the migration of the motion at the distal or proximal ends of the limb itself. In this way, the characteristics of the elbow motion can be estimated intuitively based on the shape and alignment relative to each of the limb segments. From a mechatronic perspective, we use position and orientation data to control the end effector of a robotic arm. From that application, identifying the joint variables that generate that desired position and orientation will ultimately control the end effector. However, human movement control is continuous and processed concurrently with afferent and efferent inherent modulation.

Limitations of this study are that one IMU sensor was used during the activity of a single subject. The optimal system for joint biomechanics should be characterized using two IMUs where each sensor is worn at the proximal and distal segments containing the target joint. However, previous studies have validated the use of a single IMU to measure joint angles in children with cerebral palsy [26] and to assess lower arm movements [27]. Future research will focus on increasing the use of IMUs when defining limb movements while studying the model performance in clinical and laboratory settings. The use of a single subject is also a limitation of this study. However, a robotic arm validation was also performed to address the single-subject limitation, and our study does not make a conclusion regarding the population of subjects.

By using the traditional optical-based motion tracking system, we have characterized the concept of a “knee axis” and further the concept of “invariant [8,32]”. We found that the line of the ground reaction force (GRF) vector is very close to the knee instantaneous axis (KIA). It aligns the knee joint with the GRF such that the reaction forces are torqueless. This insight shows that locating KIA is equivalent to the dynamic alignment measurement. This method can be used for the optimal design of braces and orthoses for the conservative treatment of knee osteoarthritis. Having validated the axis-angle with the optical-based system, we applied the same approach with the imu-based system to track the occupied motion of the subject.

There are several advantages of using axis-angle representations for describing limb kinematics:

1. Global Description: Axis-angle representations allow for a global description of rigid body motion without suffering from singularities due to local coordinates. Unlike traditional Euler angles, which can result in singularities and ambiguities, axis-angle representations provide a more robust and accurate representation of limb kinematics.
2. Geometric Description: Axis-angle representations provide a geometric description of rigid motion, simplifying biomechanical analysis and facilitating the understanding of kinesthesis (the feeling of movement) in skeletal and muscle structures. This geometric description is useful for applications such as computer-aided graphics, vision, and virtual reality.
3. Quaternion Operations: Axis-angle representations can be easily converted to quaternion representations, which have well-defined operations for vector addition, multiplication, and interpolation. Quaternions offer a more efficient and accurate way to represent rotations compared to other methods.
4. Simplified Biomechanics Analysis: Axis-angle representations simplify the analysis of joint biomechanics by providing a clear and intuitive representation of joint function and ligament health. They can be used to study the instantaneous axis of rotation, which plays a crucial role in joint functionality and overall locomotion perception and motor control.

Overall, using axis-angle representations for describing limb kinematics offers advantages in terms of accuracy, robustness, and simplicity of analysis.

The invariant combination of the axis-angle representation could open a new era of quantifying biomechanical perception-action systems as interactions with the natural or built environment. The overall performance metrics of many motor activities could be extended to real-world and clinical settings within multiple spatial and temporal frameworks [33]. Further, this approach may then be extended to understanding the causal nature of biomechanical injury and disease, especially that associated with inertial kinetics and kinematics [34].

The kinesthesis, the awareness of one’s own motion, cannot be measured in a SCS. However, they have unity relative to the posture and behavior of the subject being considered. The results exert goal-directed feedback control by using the IAA to guide our motion continuously. Our assumption is that goal-directed feedback could be applied to many more rehabilitation application routines. Real-time posture correction and motion change instruction could ultimately optimize motor learning, reducing injuries caused by excessive motion and bad postures.
